# Deeply Learned Classifiers for Age and Gender Predictions of Unfiltered Faces

**DOI:** 10.1155/2020/1289408

**Published:** 2020-04-30

**Authors:** Olatunbosun Agbo-Ajala, Serestina Viriri

**Affiliations:** School of Mathematics, Statistics and Computer Science, University of Kwazulu-Natal, Westville, Durban 4000, South Africa

## Abstract

Age and gender predictions of unfiltered faces classify unconstrained real-world facial images into predefined age and gender. Significant improvements have been made in this research area due to its usefulness in intelligent real-world applications. However, the traditional methods on the unfiltered benchmarks show their incompetency to handle large degrees of variations in those unconstrained images. More recently, Convolutional Neural Networks (CNNs) based methods have been extensively used for the classification task due to their excellent performance in facial analysis. In this work, we propose a novel end-to-end CNN approach, to achieve robust age group and gender classification of unfiltered real-world faces. The two-level CNN architecture includes feature extraction and classification itself. The feature extraction extracts feature corresponding to age and gender, while the classification classifies the face images to the correct age group and gender. Particularly, we address the large variations in the unfiltered real-world faces with a robust image preprocessing algorithm that prepares and processes those faces before being fed into the CNN model. Technically, our network is pretrained on an IMDb-WIKI with noisy labels and then fine-tuned on MORPH-II and finally on the training set of the OIU-Adience (original) dataset. The experimental results, when analyzed for classification accuracy on the same OIU-Adience benchmark, show that our model obtains the state-of-the-art performance in both age group and gender classification. It improves over the best-reported results by 16.6% (exact accuracy) and 3.2% (one-off accuracy) for age group classification and also there is an improvement of 3.0% (exact accuracy) for gender classification.

## 1. Introduction

Facial analysis has gained much recognition in the computer vision community in the recent past [1–4]. Human's face contains features that determine identity, age, gender, emotions, and the ethnicity of people [5, 6]. Among these features, age and gender classification can be especially helpful in several real-world applications including security and video surveillance, electronic customer relationship management, biometrics, electronic vending machines, human-computer interaction, entertainment, cosmetology, and forensic art [7–9]. However, several issues in age and gender classification are still open problems. Age and gender predictions of unfiltered real-life faces are yet to meet the requirements of commercial and real-world applications in spite of the progress computer vision community keeps making with the continuous improvement of the new techniques that improve the state of the art [10–12].

**Figure 1:**
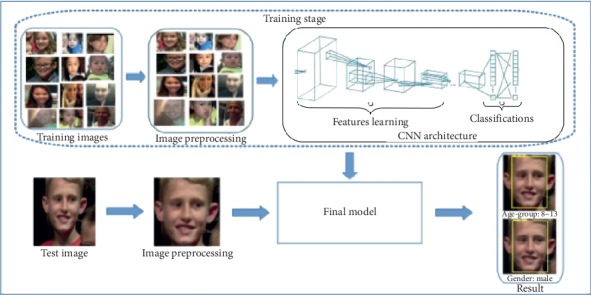
The pipeline of our framework for age group and gender.

Over the past years, a lot of methods have been proposed to solve the classifications problem. Many of those methods are handcrafted which perform unsatisfactorily on the age and gender predictions of unconstrained in-the-wild images [2, 13]. These conventional hand-engineered methods relied on the differences in dimensions of facial features and face descriptors [14–20] which do not have the ability to handle the varying degrees of variation observed in these challenging unconstrained imaging conditions. The images in these categories have some variations in appearance, noise, pose, and lighting which may affect the ability of those manually designed computer vision methods to accurately classify the age and gender of the images. Recently, deep learning-based methods [21–28] have shown encouraging performance in this field especially on the age and gender classification of unfiltered face images. In light of the current works in age and gender classification and encouraging signs of progress in deep learning and CNN, we therefore propose a novel end-to-end deep learning-based classification model that predicts age group and gender of unfiltered in-the-wild facial images. We formulate the age and gender classifications task as a classification problem in which the CNN model learns to predict the age and gender from a face image.  Figure 1 displays the overall idea of the proposed model.

The contributions of this work are summarized as follows:

1. We propose a model that uses CNN architecture to predict the age group and gender of human's faces from unfiltered real-world environments. The novel CNN approach addresses the age and gender labels as a set of discrete annotations and train the classifiers that predict the human's age group and gender.
2. We design a quality and robust image preprocessing algorithm that prepare and preprocess the unfiltered images for the CNN model and this greatly has a very strong impact on the performance accuracy of our age and gender classifiers.
3. We demonstrate that pretraining on large-scale datasets allows an effective training of our age and gender CNN model which enable the classifiers to generalize on the test images and then avoid overfitting.
4. Finally, OIU-Adience benchmark is used to evaluate the performance of our novel CNN model, and despite the very challenging nature of the images in the dataset, our approach produces significant improvements in age group and gender classification accuracy over the state-of-the-art methods; the result can satisfy the requirements of several real-world applications.

The rest of the paper is arranged as follows: in Section 2, we present related works in age and gender classification; in Section 3, we describe the proposed method for the classification task; in Section 4, we detail the experimental analysis and the results when evaluated on OIU-Adience dataset benchmark; and we summarize our contributions and conclusions with future works in Section 5.

## 2. Related Works

In this section, we briefly review the age and gender classification literature and describe both the early methods and those that are most related to our proposed method, focussing on age and gender classification of face images from unconstrained real-world environments. Almost all of the early methods in age and gender classifications were handcrafted, focussing on manually engineering the facial features from the face and mainly provides a study on constrained images that were taken from controlled imaging conditions. To mention a few, in 1999, Kwon and Lobo [29] developed the very first method for age estimation focussing on geometric features of the face that determine the ratios among different dimensions of facial features. These geometric features separate babies from adult successfully but are incapable of distinguishing between young adult and senior adult. Hence, in 2004, Lanitis et al. [30] proposed an Active Appearance Model (AAM) based method that included both the geometric and texture features, for the estimation task. This method is not suitable for the unconstrained imaging conditions attributed to real-world face images which have different degrees of variations in illumination, expression, poses, and so forth. From 2007, most of the approaches also employed manually designed features for the estimation task: Gabor [14], Spatially Flexible Patches (SFP) [17], Local Binary Patterns (LBP) [31, 32], and Biologically Inspired Features (BIF) [33]. In recent years, classification and regression methods are employed to classify the age and gender of facial images using those features. Classification methods in [12, 34–36] used Support Vector Machine (SVM) based methods for age and gender classification. Linear regression [20, 37], Support Vector Regression (SVR) [38], Canonical Correlation Analysis (CCA) [39], and Partial Least Squares (PLS) [40] are the common regression methods for age and gender predictions. Dileep and Danti [41] also proposed an approach that used feed-forward propagation neural networks and 3-sigma control limits approach to classify people's age into children, middle-aged adults, and old-aged adults. However, all of these methods are only suitable and effective on constrained imaging conditions; they cannot handle the unconstrained nature of the real-world images and, therefore, cannot be relied on to achieve respectable performance on the in-the-wild images which are common in practical applications [12].

More recently, an expanding number of researchers start to use CNN for age and gender classification. It can classify the age and gender of unfiltered face images relying on its good feature extraction technique [23, 26–28, 42–44]. Availability of sufficiently large data for training and high-end computer machines also help in the adoption of the deep CNN methods for the classification task. CNN model can learn compact and discriminative facial features, especially when the volume of training images is sufficiently large, to obtain the relevant information needed for the two classifications. For example, in 2015, Levi et al. [13] proposed a CNN based model, comprising of five layers, three convolutional and two fully connected layers, to predict the age of real-world face images. The model included centre-crop and oversampling method, to handle the small misalignment in unconstrained images. Yi et al. [45], in their paper, applied an end-to-end multitask CNN system that learns a deeper structure and the parameters needed, to solve the age, gender, and ethnicity classification task. In [46], the authors investigated a pretrained deep VGG-Face CNN approach, for automatic age estimation from real-world face images. The CNN based model consists of eleven layers, including eight convolutional and three fully connected layers. The authors in [2] also proposed a novel CNN based method, for age group and gender estimation: Residual Networks of Residual Networks (RoR). The model includes an RoR architecture, which was pretrained on gender and weighted loss layer and then on ImageNet dataset, and finally it was fine-tuned on IMDb-WIKI-101 dataset. Ranjan et al. in [47] presented a model that simultaneously solves a set of face analysis tasks, using a single CNN. The end-to-end solution is a novel multitask learning CNN framework, which shares the parameters from lower layers of CNN among all the tasks for gender recognition, age estimation, etc. In [9], the authors proposed a CNN solution for age estimation, from a single face image. The CNN based solution includes a robust face alignment phase that prepares and preprocesses the face images before being fed to the designed model. The authors also collected large-scale face images, with age and gender label: IMDb-WIKI dataset. In 2018, Liu et al. [48] developed a CNN based model that employed a multiclass focal loss function. The age estimation model was validated on Adience benchmark for performance accuracy, and it achieved a comparable result with state-of-the-art methods. Also in [49], Duan et al. introduced a hybrid CNN structure for age and gender classification. The model includes a CNN and Extreme Learning Machine (ELM). The CNN extracts the features from the input images while ELM classifies the intermediate results. In [50], the authors proposed a robust estimations solution (CNN2ELM) that also includes a CNN and ELM. The model, an improvement of the work in [49], is three CNN based solutions for age, gender, and race classification from face images. The authors in [51] also proposed a novel method based on “attention long short-term memory (LSTM) network” for age estimation in-the-wild. The method was evaluated on Adience, MORPH-II, FG-NET, LAP15, and LAP16 datasets for performance evaluation. Also in [52], the authors studied an age group-n encoding CNN based model: AGEn. The model explores the relationship between the real age and its adjacent ages, by grouping adjacent ages into the same group.

Unfortunately, some of these methods mentioned above have been verified effectively on constrained imaging conditions; few studied the unconstrained imaging conditions. However, it is still a challenging problem classifying unconstrained faces with large variations in illumination, viewpoint, nonfrontal, etc. There is a need for a suitable and robust model that can improve the state-of-the-art methods for its applicability in intelligent and real-world applications. Here, we address those issues by designing a robust image preprocessing algorithm, pretrain the model on large-scale facial aging benchmarks with noisy age and gender labels, and also regularize the CNN parameters with our novel CNN framework.

## 3. Proposed Method

This section presents the proposed deeply learned classifiers for age group and gender classification of unfiltered real-life face images, an extension of our conference paper in [53].

The approach as presented in Algorithm 1 requires image preprocessing (face detection, landmark detection, and face alignment) stage that preprocess and prepare the face images before they are input into the proposed network. Therefore, our solution is divided into three major steps: image preprocessing, features learning, and classification itself.

### 3.1. Image Preprocessing

**Figure 2:**
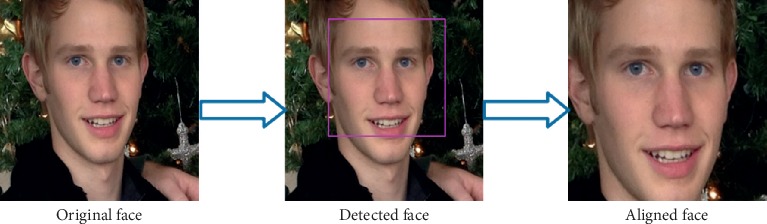
The image preprocessing phase.

Intelligent age and gender classifiers tackle the classification task under unfiltered real-world settings. Most of those face images are not aligned and nonfrontal and also with different degrees of variations in pose, appearance, lighting, and background conditions. Therefore, those in-the-wild face images need first to be detected, then aligned, and, finally, used as input for the classifiers. The image preprocessing phase as shown in Figure 2 is explained in more details below.

### 3.2. Face Detection

The first stage of image preprocessing is face detection. The face detection phase locates the face in an input image. In this work, we employ an open-source face detector: Head Hunter described in [54]. In order to detect a face, all the input images are rotated in the range of −90° to 90° angles and with the step of 5°. After that, the detector selects the input image with the best output of the face detector and in a case where the face is not detected in all the modifications of the input image, the original input image is upscaled and face detection algorithm is repeated until a face is detected. The upscaling helps in detecting faces in all the input images.

### 3.3. Landmark Detection and Face Alignment

After face detection, is the facial landmark detection and face alignment phase, where we employ the state-of-the-art solution in [55]. This image preprocessing solution is an open-source multiview facial landmark detection algorithm that uses five landmark detection models, including a frontal model, two half-profile models, and two full profile models. All of these five models are trained to work on one of the corresponding facial poses. The face alignment phase, on the other hand, entails running of all the five facial landmark models, on the detected faces. An affine transformation is then performed on the model, with the highest confidence score, to the predefined optimal settings of those landmarks.

### 3.4. CNN Architecture

Our CNN architecture is a novel six-layer network, comprising four convolutional and two fully connected layers. The CNN design is an end-to-end sequential deep learning architecture, including feature extraction and classification phases. The feature extraction phase has four convolutional layers, with the corresponding parameters, including the number of filters, the kernel size of each filter, and the stride. It contains the convolutional layer, activation layer (rectified linear unit (ReLU)), batch normalization (instead of the conventional Local Response Normalization), max-pooling layer, and a dropout [56] (all the convolutional layers have a fixed dropout of 25%). The classification stage, on the other hand, contains two fully connected layers, that handle the classification phase of the model. The first fully connected layer contains 512 neurons, followed by a ReLU, then batch normalization, and, finally, a dropout layer at a dropout ratio of 0.5. The second and the last fully connected layer output 512 features which are densely mapped to 8 or 2 neurons for classification tasks. The details of the CNN structure are presented in Table 1.

We model age group and gender classification task as an end-to-end deep classification problem; therefore, a softmax with cross entropy loss function is adopted to obtain a probability for each age group and gender class. We present the design of some parameter of the CNN architecture in the section below.

Softmax classifier gives you probabilities for each class label and its function is defined as follows:

(1) $$\begin{array}{c} f_{j}\left(s\right)=\frac{e^{s_{j}}}{\sum_{k}e^{s_{k}}}, \end{array}$$

where we are using the notation *f*_*j*_ to mean the *j*th element of the vector of class scores *f* that takes a vector of arbitrary real-valued scores in *s*.

A cross entropy loss is used for training the multiclass and binary classifications of age and gender classifiers.

We define the cross entropy for binary classification as follows:

(2) $$\begin{array}{c} H_{p}\left(q\right)=-\frac{1}{N}\sum_{i=1}^{N}\left[x_{i}\log\left(p\left({\dot{x}}_{i}\right)\right)+\left(1-x_{i}\right)\log\left(1-p{\dot{x}}_{i}\right)\right], \end{array}$$

where *x* is the binary class label, 1 if it is the correct class and 0 otherwise, and *p*(*x*) is the predicted probability of the point being green for all *N* points.

For multiclass, cross entropy is defined as follows:

(3) $$\begin{array}{c} H_{x^{i}}\left(x\right)≔-\sum_{i}x_{i}^{\prime }\log\left(x_{i}\right), \end{array}$$

where *x*_*i*_ is the predicted probability value for class *i* and *x*_*i*_′ is the true probability for that class.

### 3.5. Training Details

In this section, we describe the training details for age group and gender classifiers on IMDb, MORPH-II, and OIU-Adience datasets benchmark. The age group classifier will be responsible for predicting the age groups of unfiltered human's face images into eight different classes, while gender classifier will classify those face images into two gender classes.

For all our experiments, we initialized and trained our CNN model from scratch, using the images and the labels of IMDb and MORPH-II datasets benchmark. We primarily pretrained the novel CNN architecture on the IMDb-WIKI unfiltered facial aging dataset whose images are obtained directly from the website with some degree of variability and then fine-tuned the CNN on the images from the MORPH-II dataset, to avoid overfitting and also to adapt the CNN model to face image contents of the task to perform. Finally, we tuned the network on the training part of the actual dataset (OIU-Adience) on which we evaluated. The fine-tuning allows the CNN to pick up the distribution, the particularities, and the bias of each dataset, hence improving the performance. For each of the datasets of IMDb-WIKI and MORPH-II, we split it into two: 90% for training and 10% for validation. On the original OIU-Adience dataset, training and testing for both age and gender classification are performed using the standard 5-fold cross-validation procedure that is defined in recent literature.

### 3.6. Age Group Classification

To train the age group CNN based classifier to predict unfiltered face images into the correct age group and after series of empirical experiments, we set the initial learning rate to be 0.0001 to allow model train longer and then use an *L*2 weight decay of 0.0005. To make our model able to generalize and predict correctly, we apply Adam optimizer [57] to update network weights during training.

We present the updated equations (see equations (4)–(6)) for Adam optimization algorithm as follows:

For each parameter *w*^*j*^

(4) $$\begin{array}{c} m_{t}=\gamma_{1}*m_{t-1}+\left(1-\gamma_{1}\right)*g_{t}, \end{array}$$

(5) $$\begin{array}{c} \Delta w_{t}=-\eta \frac{v_{t}}{\sqrt{s_{t\;}+\varepsilon }}*g_{t}, \end{array}$$

(6) $$\begin{array}{c} w_{t+1}=w_{t}+\Delta w_{t}, \end{array}$$

where *η* is the learning rate, *g*_*t*_ is the gradient at time *t* along  *w*^*j*^, *m*_*t*_ and *v*_*t*_ are the exponential average of squares of gradients along  *w*_*j*_, and *γ*_1_ and *γ*_2_ are the hyperparameters.

### 3.7. Gender Classification

Training the gender classifier, we set the learning rate to an initial value of 0.01, *L*2 weight decay to 0.0005, and momentum term to 0.9. In this case, we implement a faster and popular optimization algorithm: Stochastic Gradient Descent (SGD), to update all the training models. SGD produces an update to the parameter of each training input *x*^(*i*)^ and label *y*^(*i*)^.

SGD is defined as follows:

(7) $$\begin{array}{c} \gamma =\gamma -\eta \cdot \Delta_{\gamma }J\left(\gamma ;x^{\left(i\right)};y^{\left(i\right)}\right), \end{array}$$

where *η* is defined as the learning rate and Δ_*γ*_*J* is the gradient of the loss term with respect to the weight vector *γ*.

A summary of the training details is presented in Table 2.

## 4. Proposed Algorithm

The proposed algorithm for CNN model is given in Algorithm 1.

## 5. Experiments

In this section, we first introduce the datasets with a description of their specifications and present the experimental analysis and results of the experiments when evaluated for age group and gender classifications accuracy.

### 5.1. Datasets

**Figure 3:**
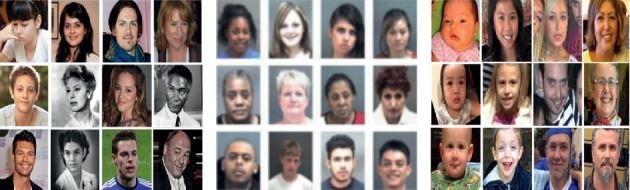
Sample face images from IMDb-WIKI, MORPH-II, and OIU-Adience datasets.

For the age group and gender classification, we evaluate the proposed method on OIU-Adience [12] dataset. IMDb-WIKI [2] and MORPH-II [58] datasets are also employed to pretrain our network when evaluating the classifiers on OIU-Adience dataset. A brief summary of those datasets is given in Table 3, with the size of each dataset, age-range information, and the number of subjects.  Figure 3 also presents some sample images of each dataset. Here, we take a brief introduction of those datasets and their specifications.

### 5.2. IMDb-WIKI [2]

IMDb-WIKI is the largest publicly available dataset for age estimation of people in the wild, containing more than half a million images with accurate age labels between 0 and 100 years. The images were crawled from IMDb and Wikipedia. IMDb contains 460,723 images of 20,284 celebrities and Wikipedia contains 62,328 images obtained directly from the website, as such the dataset contains many low-quality images, such as human comic images, sketch images, severe facial mask, full body images, multiperson images, and blank images.

### 5.3. MORPH-II [58]

MORPH-II is the mostly used dataset for real age estimation. It is a publicly available facial aging benchmark with about 55,000 facial images from more than 13,000 subjects. MORPH-II comprises 46,645 images of males and 8487 images of females with an age range from 16 to 77 years.

### 5.4. OIU-Adience [12]

**Figure 4:**
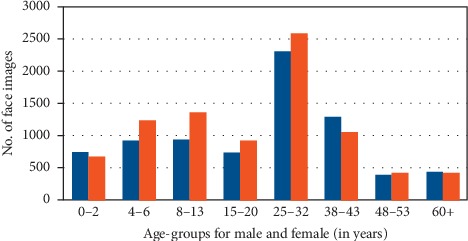
The number of image samples for age group and gender in OIU-Adience dataset.

OIU-Adience is a collection of face images from ideal real-life and unconstrained environments. It reflects all the features that are expected from an image collected from challenging real-world scenarios. They are face images that were uploaded to Flickr website from smartphone without any filtering. Adience images, therefore, display a high-level of variations in noise, pose, and appearance, among others. The entire collection of OIU-Adience dataset is about 26,580 face images of 2,284 subjects and with an age group label of eight comprising 0–2, 4–6, 8–13, 15–20, 25–32, 38–43, 48–53, and 60+. The distribution of the face images for age group and gender class labels in OIU-Adience benchmark is presented in Figure 4.

### 5.5. Experimental Results and Analysis

In this section, we present the results of our experiments on the validation set of the OIU-Adience dataset describing the impacts of our design choices on the age group and gender classification performance. We employ two different evaluating metrics common in the literature, to assess the performance of the two classifiers: exact accuracy and one-off accuracy.

### 5.6. Exact Accuracy

Exact accuracy [13] calculates the exact age group and gender results. It measures the percentage of face images that were classified into correct age group and gender, which is the ratio of the accurate predictions to the total number of the ground-truth labels. Mathematically, the metric is described as follows:

(8) $$\begin{array}{c} \text{Exact accuracy}=\frac{\text{No}.\text{ of accurate prediction}}{\text{Total no. of prediction}}. \end{array}$$

### 5.7. One-Off Accuracy

One-off accuracy [12] is off by one adjacent age group and measures whether the ground-truth class label matches the predicted class label or if the ground-truth label exists in the two adjacent bins.

We present the result for age group classification using the two criteria while the result for gender classification will be presented using exact accuracy metric.

### 5.8. Age Group Classification

**Figure 5:**
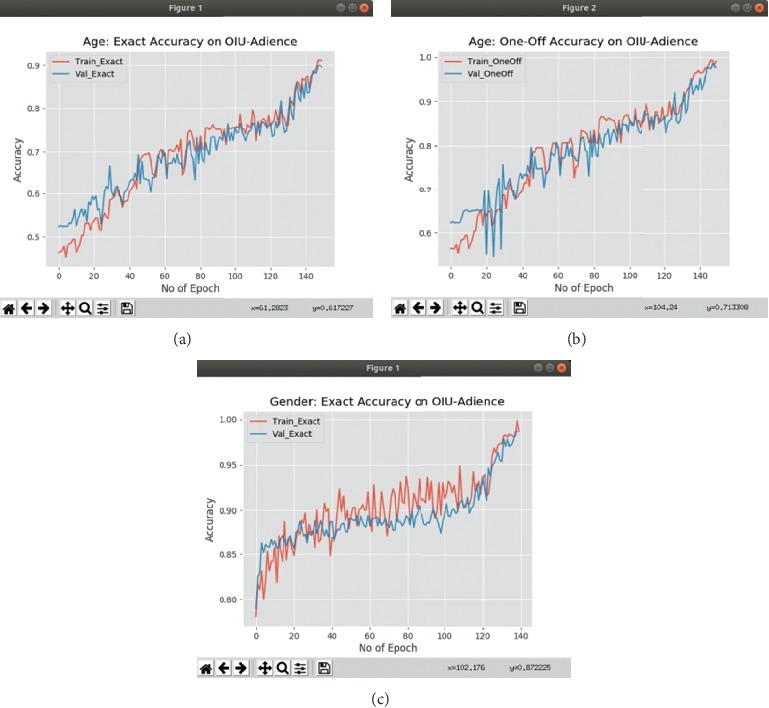
Graphs of accuracy results for the age group and gender classification. (a) Exact accuracy for age classifier. (b) One-off accuracy for age classifier. (c) Exact accuracy for gender classifier.

We evaluate our method for classifying a person to the correct age group. We train our network to classify face images into eight age group classes and report the performance of our classifier on OIU-Adience dataset, a standard dataset benchmark for the existing methods for age group and gender classification. Our model, when evaluated on the dataset, obtains an exact accuracy of 83.1% and one-off accuracy of 93.8%. This improves over the best-reported state-of-the-art result for exact accuracy in Duan et al. [50] by 16.6%, and an improvement of 3.2% on one-off result was reported in Qawaqneh et al. [46]. The graphs in Figures 5(a) and 5(b) present the exact and one-off results for the age group classification, on the validation set of OIU-Adience dataset.

### 5.9. Gender Classification

**Figure 6:**
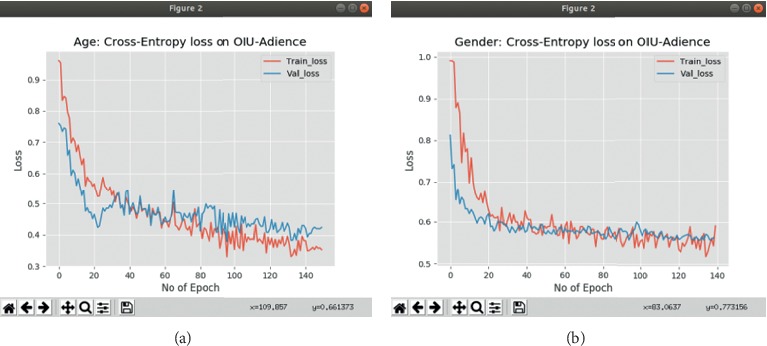
Graphs of cross entropy loss results for the age group and gender classification. (a) Cross entropy loss for age classifier. (b) Cross entropy loss for gender classifier.

We also evaluate our method for classifying a person to the correct gender. We assess the performance on the same Adience dataset consisting of labels for gender. For this task, we train our network for classification of two classes and report the result on exact accuracy, with pretraining on the three datasets. As presented in Figure 5(c), we achieve an accuracy of 96.2% compared to the previous state-of-the-art of 93.2% in Zhang et al. [2] showing an improvement of 3.0%. Our approach, therefore, achieves the best results not only on the age group estimation but also on gender classification; it outperforms the current state-of-the-art methods. Tables 4 and 5 present the classification results of the best configuration of our method with that of the state-of-the-art methods on OIU-Adience benchmark dataset. All the reported results have been computed using the same 5-fold cross-validation protocol. We also present the loss function results for the two classifications in Figures 6(a) and 6(b).

In the first line of Table 6, we present the results for the age group and gender classifications, when pretrained on IMDb-WIKI dataset. These results are to be compared with the results in line 2 which represent the performance of our model fine-tuned on the training set of another large facial aging dataset (MORPH-II). A huge improvement in the results from line 1 shows clearly the importance of fine-tuning our CNN model on huge training images with age and gender annotations, to learn from the representation power of the CNN and pick up the bias and peculiarities of the dataset. Line 3 of Table 6, highlights the impact of the quality of a robust image preprocessing algorithm, on the classification accuracy. The data augmentation during the fine-tuning on the original (OIU-Adience) dataset (line 4 of Table 6) proved to be very efficient as well, with an improvement of the results from line 3 (fine-tuning without data augmentation). Finally, the last line of Table 6 proves the importance of utilizing the Batch Normalization (BN) rather than the conventional and deprecated Local Response Normalization (LRN); this improved the classification accuracy of our model.

**Figure 7:**
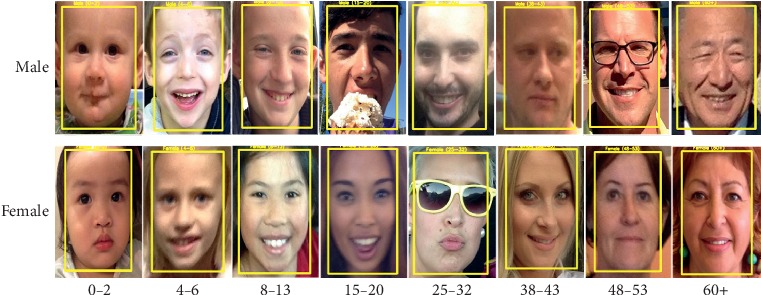
Samples of face images with correct age group and gender classifications.

**Figure 8:**
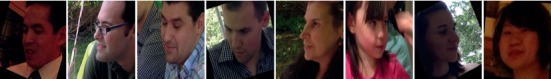
Samples of face images with misclassified age group or gender.

Some samples of validation classification results are shown in Figure 7, where we present the age group and gender predictions of some of the face images from the OIU-Adience dataset using the newly designed model. In many cases, our solution is able to correctly predict the age group and gender of faces. Undoubtedly, our proposed model did result in some cases of age group and gender misclassification. Some of those mi-classification results are presented in Figure 8. The classification failure could be as a result of the extremely challenging viewing conditions of the OIU-Adience images including low resolution, lighting conditions, and heavy makeup, hence hindering our image preprocessing algorithm in either correctly detecting or aligning the face for the classification process.

## 6. Conclusion and Future Works

We tackled the classification of age group and gender of unfiltered real-world face images. We posed the task as a multiclass classification problem and, as such, train the model with a classification-based loss function as training targets. Our proposed model is originally pretrained on age and gender labelled large-scale IMDb-WIKI dataset, whose images are obtained directly from the website with some degree of variability and then fine-tuned on MORPH-II, another large-scale facial aging dataset with age and gender annotations. Finally, we use the original dataset (OIU-Adience benchmark of unfiltered faces for age and gender classification) to fine-tune this model. The robust image preprocessing algorithm, handles some of the variability observed in typical unfiltered real-world faces, and this confirms the model applicability for age group and gender classification in-the-wild. Finally, we investigate the classification accuracy on OIU-Adience dataset for age and gender; our proposed method achieves the state-of-the-art performance, in both age group and gender classification, significantly outperforming the existing models. For future works, we will consider a deeper CNN architecture and a more robust image processing algorithm for exact age estimation. Also, the apparent age estimation of human's face will be interesting research to investigate in the future.

## Figures and Tables

**Algorithm 1:**
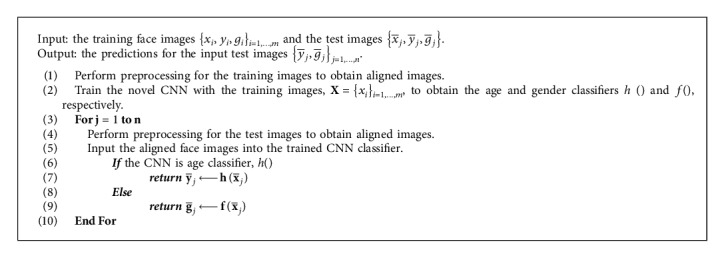
Algorithm for the age and gender classifiers.

**Table 1:** The details of CNN architecture for the proposed method.

Layer type	Output size	Filter size/stride
Input image	227 × 227 × 3	—
CONV1	56 × 56 × 96	7 × 7/4 × 4
ACT	56 × 56 × 96	—
BN	56 × 56 × 96	—
Maxpool	28 × 28 × 96	3 × 3/2 × 2
Dropout	28 × 28 × 96	—
CONV2	28 × 28 × 256	5 × 5
ACT	28 × 28 × 256	—
BN	28 × 28 × 256	—
Maxpool	14 × 14 × 256	3 × 3
Dropout	14 × 14 × 256	—
CONV3	14 × 14 × 384	3 × 3
ACT	14 × 14 × 384	—
BN	14 × 14 × 384	—
Maxpool	7 × 7 × 384	3 × 3
Dropout	7 × 7 × 384	—
CONV4	7 × 7 × 384	3 × 3
ACT	7 × 7 × 256	—
BN	7 × 7 × 256	—
Maxpool	1 × 1 × 256	3 × 3
Dropout	1 × 1 × 256	—
FC1	512	—
ACT	512	—
BN	512	—
Dropout	512	—
FC2	2 or 8	—

**Table 2:** Training details with OIU-Adience.

Classifier	Optimizer	No. of epochs	Initial learning rate	Momentum term	*L*2 weight decay
Age group	Adam	150	0.0001	—	0.0005
Gender	SGD	140	0.01	0.9	0.0005

**Table 3:** The details of the datasets used in our experiments.

Dataset	Dataset size	No. of subjects	Age type	Age range
IMDb-WIKI [2]	523,051	20,284	Real age	0–100
MORPH-II [58]	55,134	13,618	Real age	16–77
OIU-Adience [12]	26,580	2284	Age group	0–60+

**Table 4:** Age group classification: performance comparison of our result with the state-of-the-art works on OIU-Adience dataset.

Methods	Exact accuracy (%)	One-off (%)
[12]	45.1	79.5
[13]	50.7	84.7
[59]	58.5	—
[46]	59.9	90.6
[49]	52.3	—
[48]	54.0	88.2
[50]	66.5	—
*Proposed*	**83.1**	**93.8**

**Table 5:** Gender classification: performance comparison of our result with the state-of-the-art works on OIU-Adience dataset.

Methods	Exact accuracy (%)
[13]	86.8
[2]	93.2
[49]	88.2
*Proposed*	**96.2**

**Table 6:** The details of experimental results of our model on OIU-Adience dataset.

Pretraining on IMDb-WIKI	Fine-tuning on MORPH-II	Image preprocessing	Data augmentation	Batch normalization	Exact acc. (age)	One-off (age)	Exact acc, (gender)
Yes	No	No	No	No	71.2	84.8	91.3
Yes	Yes	No	No	No	76.1	88.3	93.8
Yes	Yes	Yes	No	No	79.3	90.6	94.5
Yes	Yes	Yes	Yes	No	81.2	91.8	95.9
Yes	Yes	Yes	Yes	Yes	**83.1**	**93.8**	**96.2**

## Data Availability

No data were used to support this study.
